# Soybean β-Conglycinin Prevents Age-Related Hearing Impairment

**DOI:** 10.1371/journal.pone.0137493

**Published:** 2015-09-08

**Authors:** Tohru Tanigawa, Rei Shibata, Kazuhisa Kondo, Nobuyuki Katahira, Takahiro Kambara, Yoko Inoue, Hiroshi Nonoyama, Yuichiro Horibe, Hiromi Ueda, Toyoaki Murohara

**Affiliations:** 1 Department of Otolaryngology, Aichi Medical University, Aichi, Japan; 2 Department of Cardiology, Nagoya University Graduate School of Medicine, Nagoya, Japan; AntiCancer Inc., UNITED STATES

## Abstract

Obesity-related complications are associated with the development of age-related hearing impairment. β-Conglycinin (β-CG), one of the main storage proteins in soy, offers multiple health benefits, including anti-obesity and anti-atherosclerotic effects. Here, to elucidate the potential therapeutic application of β-CG, we investigated the effect of β-CG on age-related hearing impairment. Male wild-type mice (age 6 months) were randomly divided into β-CG-fed and control groups. Six months later, the body weight was significantly lower in β-CG-fed mice than in the controls. Consumption of β-CG rescued the hearing impairment observed in control mice. Cochlear blood flow also increased in β-CG-fed mice, as did the expression of eNOS in the stria vascularis (SV), which protects vasculature. β-CG consumption also ameliorated oxidative status as assessed by 4-HNE staining. In the SV, lipofuscin granules of marginal cells and vacuolar degeneration of microvascular pericytes were decreased in β-CG-fed mice, as shown by transmission electron microscopy. β-CG consumption prevented loss of spiral ganglion cells and reduced the frequencies of lipofuscin granules, nuclear invaginations, and myelin vacuolation. Our observations indicate that β-CG ameliorates age-related hearing impairment by preserving cochlear blood flow and suppressing oxidative stress.

## Introduction

Age-related hearing impairment (ARHI) is common in older populations, with an estimated prevalence of 30–60%; it has a strong influence on quality of life [1–3]. Recently, higher body mass index (BMI) and waist circumference have been associated with the severity of hearing loss [4,5]. Obesity-related disorders including dyslipidemia and type 2 diabetes are also associated with ARHI [6–8]. Thus, management of lifestyle-related diseases plays a pivotal role in the prevention of hearing impairment.

β-Conglycinin (β-CG) is one of the major storage proteins in soy and is composed 30–40% of soy protein [9]. Recent experimental studies have shown that β-CG in foods could be used to prevent lifestyle-related disease such as obesity and dyslipidemia [10,11]. β-CG reduces plasma cholesterol and triglycerides in rats and exerts blood pressure-lowering effects in spontaneous hypertensive rats [12,13]. It is also more effective than whole soy protein in reducing atherosclerosis in experimental mouse models of the disease [14]. Consistent with these observations, the consumption of β-CG for 4 weeks reduced triglyceride levels in patients with hyperlipidemia and was shown to reduce high visceral fat and body-fat ratios in human subjects [15]. However, the effects of β-CG on hearing impairment have not been examined. In this study, we sought to determine whether β-CG consumption prevents early sensorineural hearing loss in mice.

## Materials and Methods

### Materials

eNOS antibodies were purchased from Sigma (St. Louis, MO). 4-hydroxynonenal (4-HNE) antibody was purchased from Alpha Diagnostic International Inc. (San Antonio, TX). β-CG was provided as a generous gift by Fuji Oil (Osaka, Japan). Vitamin-free casein was purchased from Oriental Yeast (Tokyo, Japan).

### Animals and experimental protocol

Six-month-old male C57BL/6J wild-type (WT) mice were used according to protocols approved by the Institutional Animal Care and Use Committee of Aichi Medical University (No. 2014-1). The mice were randomly divided into a β-CG-fed group and a casein-fed control group for the 6-month-long experiment. Experimental diet compositions are shown in Table 1. Treatment with β-CG was initiated in 6-month-old mice and continued for the indicated duration of time. Mice were maintained on a 12 h/12 h light/dark cycle in an atmosphere-controlled room. Body weight was recorded at 6, 8, and 12 months of age.

**Table 1:** Composition of experimental diets.

Ingredient (g/100 g)	Control	β-CG
Casein	22.7	-
β-Conglycinin	-	21.6
Cornstarch	37.35	38.45
Dextrinized cornstarch	13.2	13.2
Sucrose	10	10
Soybean oil	7	7
Cellulose powder	5	5
Mineral mixture	3.5	3.5
Vitamin mixture	1	1
Choline bitartrate	0.25	0.25

### Auditory brainstem response measurement

At 6, 8, and 12 months, the mice were subjected to ABR measurement under anesthesia with ketamine (100 mg/kg) and xylazine (9 mg/kg) as previously described [16]. In brief, the mice were placed in a quiet room, and generation of acoustic stimuli and subsequent recording of evoked potentials were performed using a data acquisition system (AD Instruments, NSW, Australia). Acoustic stimuli, consisting of tone burst stimuli (0.1 ms cos2 rise/fall and 1 ms plateau), were delivered monaurally through a magnetic speaker (CF1, Tucker-Davis Technologies, FL, USA) connected to a funnel and fitted into the external auditory meatus. To record bioelectrical potentials, subdermal stainless steel needle electrodes were inserted at the vertex (ground), ventrolateral to the measured ear (active), and contralateral to the measured ear. Stimuli were calibrated against a 1/4” condenser microphone (UC-54, RION, Tokyo, Japan) connected to a measuring amplifier (NA-42, RION, Tokyo, Japan). Responses between the vertex and mastoid subcutaneous electrodes were amplified with a differential extracellular amplifier (ER-1, Cygnus Technology, Southport, NC). Thresholds were determined for frequencies of 8, 16, and 32 kHz from a set of responses at varying intensities with 5-dB SPL intervals, and electrical signals were averaged at 512 repetitions.

### Analysis of cochlear blood flow

Cochlear blood flow (CBF) was measured at the age of 12 months in the mice by using a laser-Doppler flowmeter (OMEGA FLOW, FLO-C1, Neuroscience, Tokyo, Japan) as described [16]. The left bulla of each mouse was identified and opened carefully using a ventrolateral approach. After removing the mucosa and periosteum overlying the bone, a probe (GJ type) from a laser-Doppler flowmeter was placed over the basal turn of the cochlea, where the maximum output of CBF was measured in arbitrary units (AU). CBF values (mean for 60 sec after stabilization) were collected using a computer-based chart-recorder.

### Histology

Mice were perfused intracardially with physiological saline, followed by 4% paraformaldehyde in 0.01 M phosphate-buffered saline (PBS) at pH 7.4. Excised temporal bones were immersed in the same fixative at 4°C for 4 h. The cochleae were dissected from the temporal bones in PBS. Cochlear specimens were then placed in 0.1 M ethylenediaminetetraacetic acid in PBS for decalcification. The samples were incubated with 30% sucrose in PBS at 4°C prior to embedding in OCT compound (Tissue-Tek, Sakura Finetechnical, Tokyo, Japan), and frozen at -80°C. Mid-modiolus sections (7 μm in thickness) were sliced and stained with hematoxylin and eosin [16]. To measure eNOS in the stria vascularis (SV), we stained tissue sections with primary antibodies (dilution1:200) and biotin-conjugated secondary antibody [17]. We also evaluated reactive oxygen species (ROS) production by 4-HNE staining [18]. Frozen tissue slices were stained with 4-HNE followed by treatment with the secondary antibody system: Histofine Simple Stain MAX-PO (Nichirei Corp., Tokyo, Japan).

### Electron microscopy

Mice were perfused intracardially with physiological saline, followed by 2% glutaraldehyde plus 2% PFA in 0.1 M cacodylate buffer at pH 7.4. Temporal bones were dissected and perfused with fixative solution via the round and oval windows. The cochleae were post-fixed in 2% osmium tetroxide and dehydrated in ethanol for electron microscopy. The specimens were secondarily-stained with Lead stain solution (Sigma-Aldrich) and the grids were observed by transmission electron microscopy at an acceleration voltage of 80 kV (JEM-1200EX; JEOL Ltd.). Digital images were obtained with a CCD camera (VELETA; Olympus Soft Imaging Solutions GmbH) [19].

### Statistical analysis

All data are presented as mean ± SEM. Statistical analysis was performed by ANOVA followed by Scheffe’s method or Mann-Whitney U test. A P value of *P* < 0.05 was considered significant. All analyses were performed using JMP (version 6.03; SAS Institute Inc.).

## Results

### Effect of β-CG on body weight

**Fig 1:**
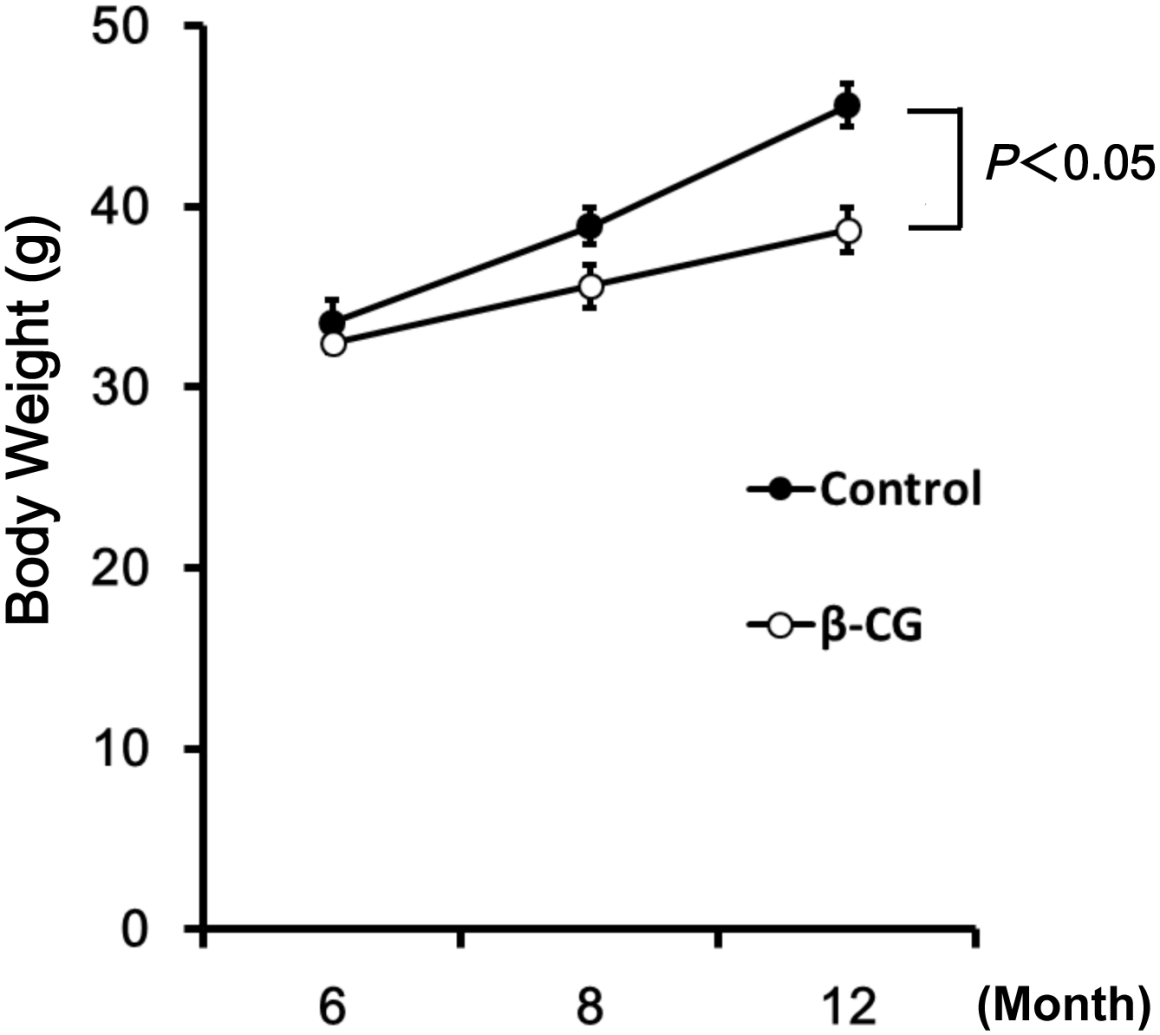
Effect of β-CG on body weight in WT mice. Body weight (BW) was similar between the two groups at 6 and 8 months of age. At 8 months, BW was higher in the controls than in the βCG-fed group, but the difference was not significant. At 12 months, BW was significantly higher in the controls than in the βCG-fed group (*P* < 0.05).

The control casein-fed mice showed a moderate 35.7% increase in body weight. At the end of the 6-month-long experiment, the average body weight of β-CG-fed mice was 38.7 ± 1.2 g, which was 15.1% lower than that of the controls (45.6 ± 1.2 g) (Fig 1). Daily food intake did not differ between the β-CG-fed and control groups.

### Consumption of β-CG prevents hearing impairment

**Fig 2:**
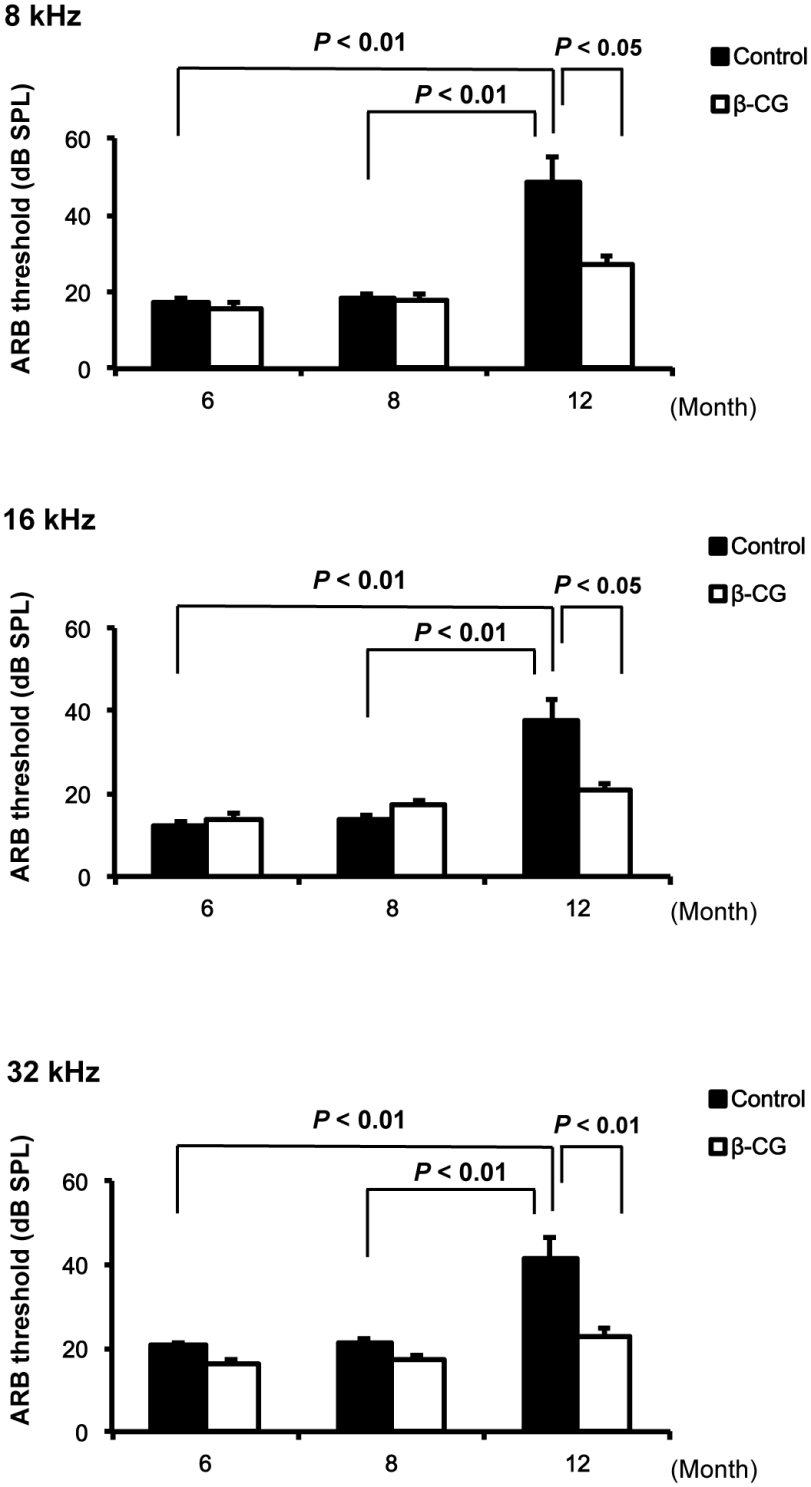
Consumption of β-CG prevents hearing impairment in mice. At 6 and 8 months, control mice showed no threshold shifts at any frequencies. β-CG consumption did not affect ABR thresholds at these ages. At the age of 12 months, control mice showed a significant increase in ABR thresholds at 8, 16, and 32 kHz. Consumption of β-CG prohibited the increase in ABR thresholds (*n* = 7 in each group).

To test whether β-CG can prevent the development of hearing impairment, we assessed ABR thresholds in β-CG-fed mice at ages of 6, 8, and 12 months (Fig 2, *n* = 7 per group). At 6 and 8 months, the control mice showed no threshold shifts at any frequencies. β-CG consumption did not affect ABR thresholds at these ages. However, at 12 months, the control mice showed a significant increase in ABR thresholds at 8, 16, and 32 kHz. Consumption of β-CG prevented the increase in ABR thresholds at 8, 16, and 32 kHz that was seen in control mice at 12 months.

### β-CG increases CBF

**Fig 3:**
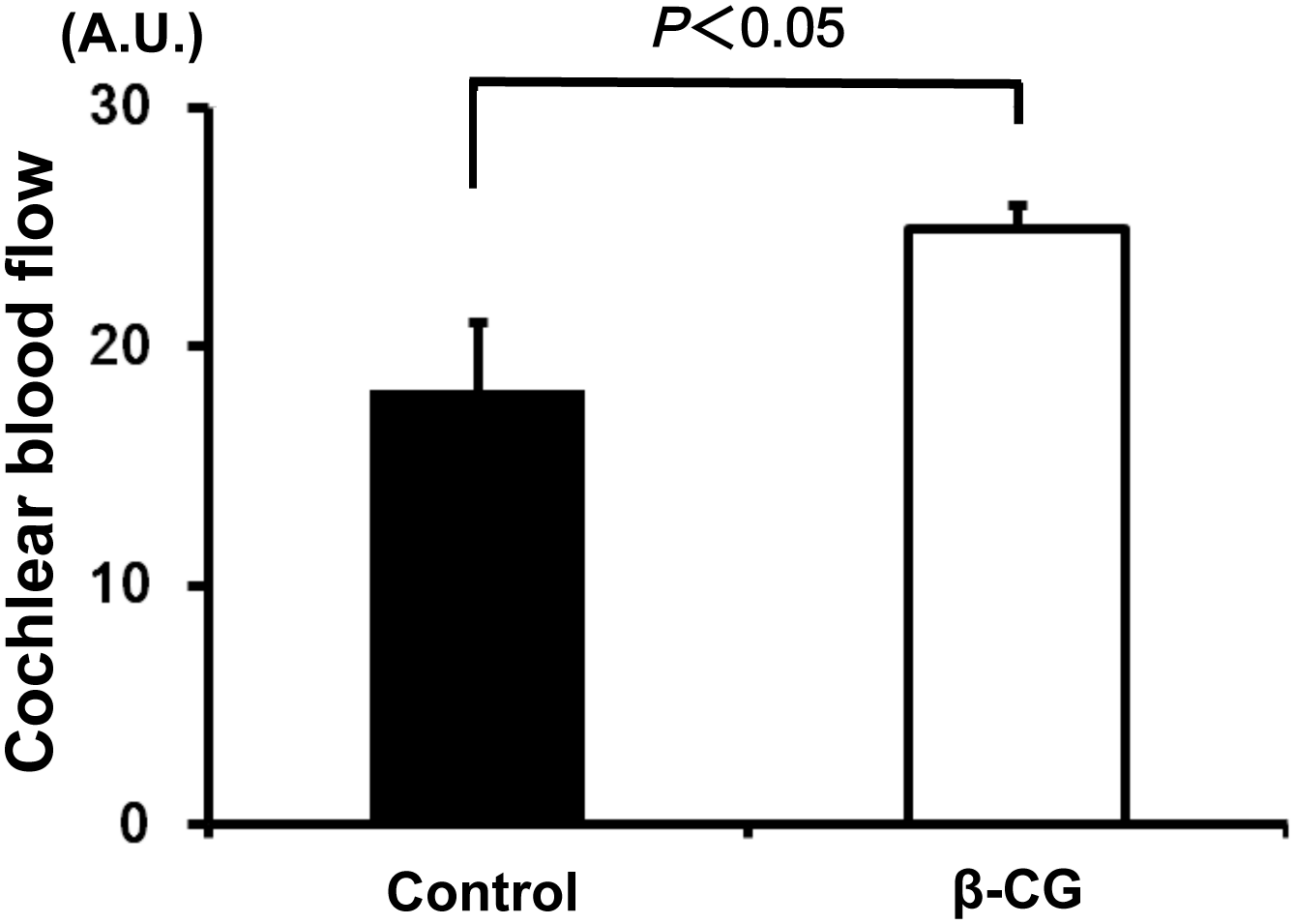
β-CG increases cochlear blood flow in WT mice. Quantitative analysis revealed that casein-fed mice showed a marked reduction in cochlear blood flow of 27.6% ± 8.5% versus βCG-fed mice (*P* < 0.05).

Because increased cochlear blood flow contributes to the prevention/treatment of hearing impairment, we measured CBF using a laser-Doppler flowmeter [20,21]. At 12 months, β-CG-fed mice showed a marked increase in CBF of 27.6% ± 8.5% versus the control mice (Fig 3, *n* = 3, *P* < 0.05).

### β-CG increases eNOS expression and reduces oxidative damage in the stria vascularis

**Fig 4:**
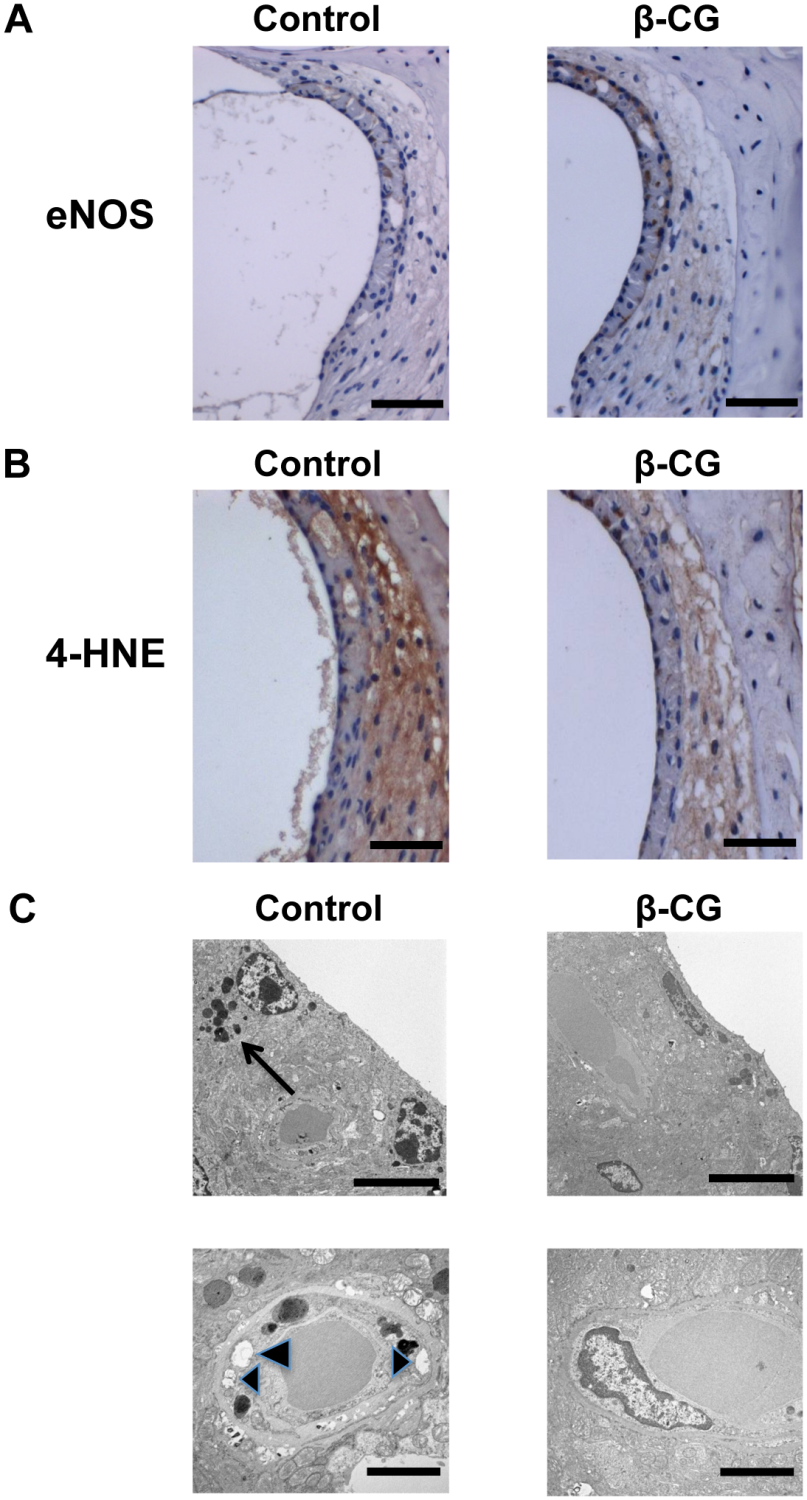
β-CG increases eNOS expression, reduces oxidative damage, and maintains microstructure in the stria vascularis. (A) Representative photomicrographs of tissue immunostained for eNOS. eNOS expression in the stria vascularis of the cochlear basal turn was higher in βCG-fed mice than in the control mice (scale bar: 50 μm). (B) Representative photographs of 4-HNE staining in the SV. Expression of 4-HNE was abundant in control mice and was reduced by β-CG consumption (scale bar: 50 μm). (C) Transmission electron microscopy revealed greater numbers of lipofuscin granules in control mice (upper-left panel, arrow) than in βCG-fed mice (scale bar: 5 μm). Vacuolar degeneration in microvascular pericytes was frequent in control mice (lower-left panel, arrowheads) (scale bar: 2 μm).

eNOS plays an important role in the vascular function in various organs including the cochlea [17,22]. To analyze the potential involvement of eNOS in β-CG-induced increase in blood flow in the cochlea, eNOS expression was assessed in histological sections from the cochlear lateral wall. Fig 4A shows representative photomicrographs of tissue immunostained for eNOS in 12-month-old mice. Expression of eNOS in the SV was increased by β-CG consumption.

Oxidative stress is responsible for the age-related damage to the organ of Corti [23,24]. To investigate the production of ROS in the cochlea, we performed 4-HNE staining in the cochlear lateral wall of 12-month-old mice. Representative photographs of 4-HNE staining in the SV are shown in Fig 4B. Expression of 4-HNE was abundant in control mice and was attenuated by β-CG consumption.

Transmission electron microscopy revealed lipofuscin granules of marginal cells and vacuolar degeneration of microvascular pericytes in the SV of 12-month-old control mice (Fig 4C), but not in the SV of the β-CG-fed mice.

### β-CG prevented the loss of spiral ganglion cells

**Fig 5:**
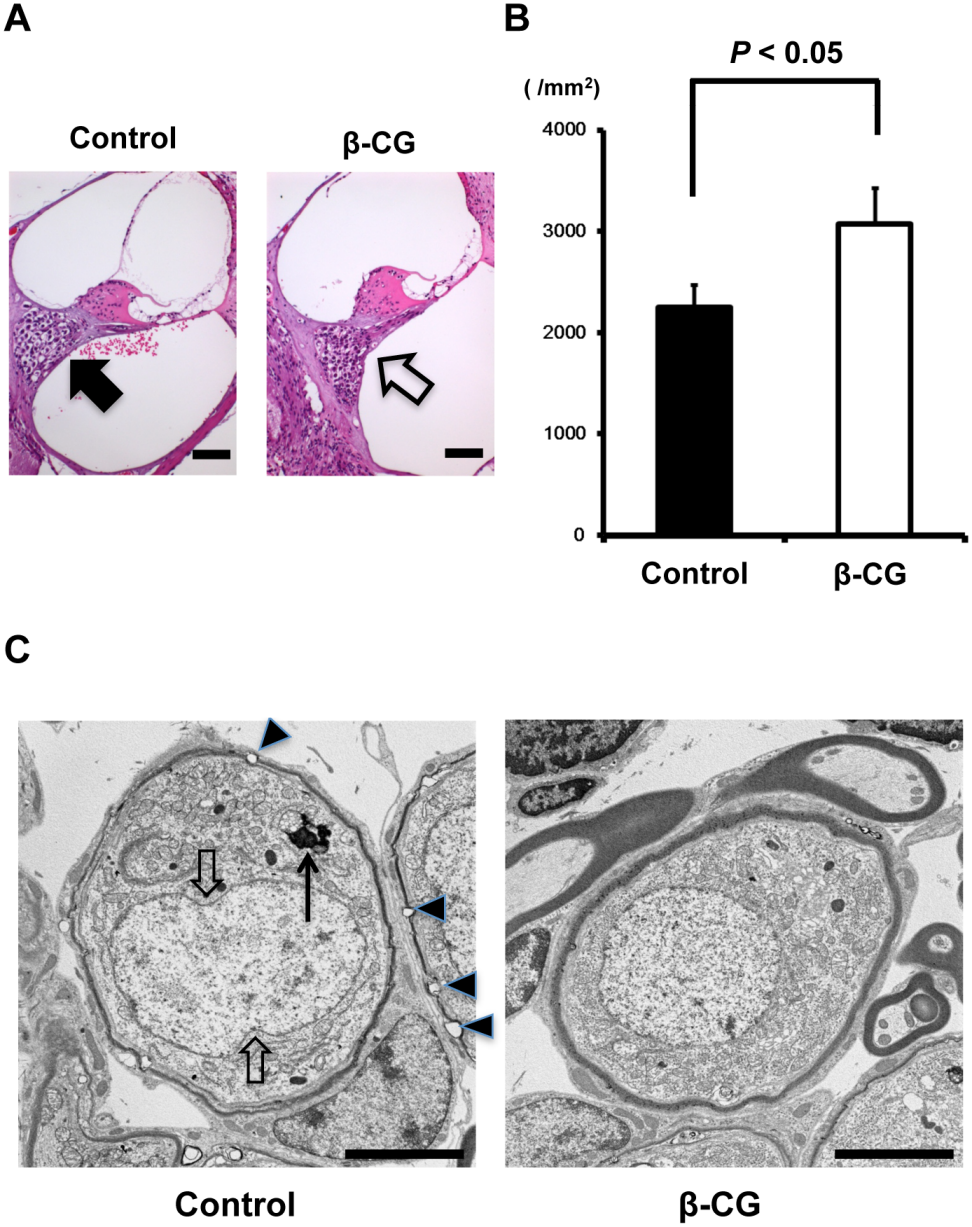
β-CG prevents loss of spiral ganglion cells. (A) Representative photomicrographs of spiral ganglion cells in the cochlear basal turns of control (arrow) and βCG-fed mice (white arrow) at 12 months of age (scale bar: 50 μm). Spiral ganglion cells were lost in control mice and preserved in βCG-fed mice. (B) Quantitative analysis revealed that the spiral ganglion cell density was significantly lower in the control than in the βCG-fed mice (*n* = 4, *P* < 0.05). (C) Transmission electron microscopy revealed that the numbers of lipofuscin granules in spiral ganglion cells was significantly higher in the control mice (long arrow) than in the βCG-fed mice (scale bar: 5 μm). Moreover, morphological damage such as nuclear invaginations (white arrow) or vacuolation in the myelin sheath (arrowheads) were also observed in control mice but were virtually absent in β-CG-fed WT mice (Fig 5C, right panel).

Degeneration of the spiral ganglion in the cochlea is a hallmark of ARHI [25]. We assessed the morphological alterations in the spiral ganglion in our experimental mice. Cochlear tissue in the basal turn was stained with hematoxylin and eosin. Control mice showed a loss of spiral ganglion cells (SGCs), which were preserved in β-CG-fed mice at 12 months (Fig 5A). Quantitative analysis revealed that the number of SGCs in the spiral ganglion was significantly reduced in the β-CG-fed mice (Fig 5B, *n* = 4, *P* < 0.05 vs. the controls).

Finally, we assessed SGCs by transmission electron microscopy. Lipofuscin granules (long arrow), nuclear invaginations (arrows), and myelin vacuolation (arrowheads) in the SGCs were observed in control mice but not in β-CG-fed WT mice (Fig 5C).

## Discussion

The major findings of this study are as follows: (1) β-CG suppressed body weight gain, (2) prevented the development of hearing impairment, (3) increased cochlear blood flow, (3) increased eNOS expression and reduced oxidative damage in SV, and finally, (4) prevented loss of spiral ganglion cells.

The β-CG-fed mice exhibited less body weight gain than control mice, despite comparable food intake. Recent epidemiological studies have associated obesity with the severity of hearing loss [4,5]. Diet-induced obesity exacerbates auditory degeneration in mice [26]. In contrast, caloric restriction slows the progression of ARHI in various strains of mice [27]. Thus, long-term weight control is an important factor for the prevention of ARHI. Intake of β-CG has been shown to decreases the body fat in humans and animals [10,11,15]. These data suggest β-CG is an important food component for the prevention of obesity-related diseases including ARHI.

Age-induced cochlear hypo-perfusion causes damage to the organ of Corti and induces hearing loss [16,20,21]. Reduced CBF and impaired hearing are observed in ApoE-KO mice and streptozotocin-induced diabetic mice [28,29]. The expression of eNOS, which protects the vasculature, is severely reduced in the cochlea of ApoE-KO mice, especially in animals fed a high-fat diet [28]. We showed that β-CG consumption leads to increased CBF in the SV. β-CG also increased eNOS expression in the SV. It is possible that β-CG increases blood flow in the cochlea, thereby contributing to the prevention of hearing impairment.

Further, oxidative stress is associated with rapid aging and correlates with hearing loss [23,24]. Supplementation with α-lipoic acid, an anti-oxidant drug, prevents ARHI in mice [30]. 4-HNE, an aldehyde product of membrane lipid peroxidation, could be produced by oxidative stimuli [31]. Immune-reactivity to 4-HNE is stronger in aged mice cochlea than in the cochlea of young mice [23]. In this study, cochlear expression of 4-HNE was attenuated by β-CG consumption. These data suggest that β-CG prevents the development of hearing impairment, in part, via improvement of the oxidative status in the cochlea.

In conclusion, our observations indicate that consumption of β-CG prevented age-related hearing impairment by preserving cochlear blood flow and improving oxidative status. The main bioactive component in soy protein may be β-CG, which can be used in nutritional supplementation in age and lifestyle-related diseases such as obesity and hearing impairment.
